# From Feedstock to Function: How Pyrolysis and Oxidation Shape Biochar Performance in Soil–Plant Interactions

**DOI:** 10.3390/plants14213278

**Published:** 2025-10-27

**Authors:** Mohammad Ghorbani, Elnaz Amirahmadi, Jaroslav Bernas, Jan Bárta

**Affiliations:** 1 School for Environment and Sustainability, University of Michigan, Ann Arbor, MI 48109, USA; mghorban@umich.edu; 2 Faculty of Agriculture and Technology, University of South Bohemia in České Budějovice, 370 05 České Budějovice, Czech Republic; bernas@fzt.jcu.cz (J.B.); barta@fzt.jcu.cz (J.B.)

**Keywords:** biochar modification, pyrolysis temperature, soil nutrient availability, soil–plant interactions, nutrient uptake

## Abstract

Nutrient losses through leaching and low nutrient use efficiency are major challenges limiting crop productivity and causing environmental pollution. Biochar has been widely studied as a soil amendment to improve nutrient retention; however, the combined effects of pyrolysis temperature and post-production oxidation on soil nutrient dynamics and plant performance remain unclear. In this study, wheat straw and wood residue biochars were produced at two pyrolysis temperatures (350 and 450 °C) and subsequently modified by hydrogen peroxide (H_2_O_2_) oxidation to enhance surface functionality. A pot experiment with fava bean (*Vicia faba* L.) was conducted to evaluate the effects of pristine and oxidized biochars on soil chemical properties, nutrient leaching, and plant nutrient uptake. Results showed that pristine biochars increased soil pH from 6.82 (control) to 8.73–9.12 and EC from 2.15 to 3.06–4.71 dS m^−1^, with wheat straw biochars having stronger alkalizing effects. In contrast, oxidized biochars decreased soil pH to 5.62–5.93 due to the introduction of oxygen-containing functional groups. All biochars reduced NO_3_^−^-N, NH_4_^+^-N, and PO_4_^3−^-P leaching, with the most pronounced reductions observed in oxidized wheat straw biochar produced at 450 °C (O-BWS_450_). Improved nutrient retention translated into higher plant nutrient uptake: fava bean plants grown in O-BWS_450_-amended soil achieved the greatest N (6.71%) and P (3.89%) uptake, significantly higher than the control. These findings highlight the potential of oxidation-modified biochars, particularly wheat straw biochar produced at moderate pyrolysis temperature, to improve soil nutrient conservation and enhance crop nutrition simultaneously. Such modifications represent a promising approach for developing biochar-based soil amendments that promote sustainable nutrient management.

## 1. Introduction

Soil degradation and nutrient depletion are among the most pressing challenges in global agriculture, threatening food security and sustainable land management [1,2]. Excessive reliance on chemical fertilizers, coupled with poor organic matter management, has led to reduced soil fertility, nutrient leaching, and environmental pollution [3]. In this context, biochar, a carbon-rich byproduct of biomass pyrolysis, has gained increasing attention as a multifunctional soil amendment capable of improving soil physicochemical properties, enhancing nutrient retention, and supporting plant growth [4,5].

Biochar’s unique properties, including its porous structure, high cation exchange capacity, and recalcitrance in soil, make it an attractive tool for restoring degraded soils and promoting sustainable nutrient cycling [6,7,8]. However, the performance of biochar in soil-plant systems is highly variable, depending largely on the feedstock type, pyrolysis temperature, and post-treatment modifications [9,10]. Importantly, biochar application can influence soil electrical conductivity (EC), which is an indicator of soil salinity [11]. Excessive increases in EC may lead to salinity stress, reducing water uptake by plants, impairing nutrient availability, and ultimately decreasing crop productivity [12,13]. Therefore, monitoring EC and managing potential salinity issues are critical when applying biochar, particularly in soils prone to salt accumulation [14,15]. Including such considerations provides context for understanding both the benefits and potential limitations of biochar in agricultural systems.

Raw or unmodified biochar may exhibit limited surface activity or nutrient retention capacity, especially when derived from feedstocks with low lignin content or produced under suboptimal thermal conditions [16,17]. The choice of feedstock—such as wood residues or agricultural byproducts like wheat straw—directly influences the chemical composition and structural integrity of the resulting biochar [18]. Moreover, the pyrolysis temperature plays a crucial role in determining the physical and chemical properties of biochar. Low-temperature pyrolysis (300–400 °C) tends to preserve more volatile organic compounds and oxygenated functional groups, while high-temperature pyrolysis (450–600 °C) generally enhances carbonization and surface area but may reduce functional group availability [19,20,21]. Despite these established trends, the ideal combination of feedstock and pyrolysis conditions is still debated and appears to vary depending on the intended application. This variability points to the need for targeted modification techniques to optimize biochar performance for specific agricultural purposes.

Recent research has focused on chemical and physical modifications of biochar to increase its reactivity and efficiency in soil applications [22,23]. Among these, hydrogen peroxide (H_2_O_2_) oxidation has emerged as a simple, cost-effective method for increasing biochar surface area, introducing more oxygen-containing functional groups (e.g., hydroxyl, carbonyl, carboxyl), and enhancing porosity [24]. These alterations may significantly improve biochar’s nutrient adsorption capacity, interaction with soil microbiota, and influence on plant nutrient uptake [25,26]. However, studies evaluating oxidized biochar under controlled soil–plant conditions remain limited, especially those systematically comparing multiple feedstocks, pyrolysis temperatures, and oxidation treatments. This gap hinders our ability to design biochar products tailored for both soil fertility improvement and plant growth promotion.

In this study, we aimed to evaluate how biochar feedstock type, pyrolysis temperature, and hydrogen peroxide (H_2_O_2_) oxidation influence the chemical and functional properties of biochar and its performance in a soil–plant system.

Specifically, we sought to (I) compare biochars derived from two common feedstocks (wood residue and wheat straw) produced at two pyrolysis temperatures (350 °C and 450 °C); (II) assess how H_2_O_2_ oxidation modifies biochar’s effects on soil chemical properties, including pH and electrical conductivity (EC); (III) quantify the impacts of these treatments on soil nutrient leaching (nitrate, ammonium, and phosphate) and plant nutrient uptake (nitrogen and phosphorus).

Through these objectives, this study provides insight into how targeted biochar engineering, via feedstock selection, thermal processing, and chemical oxidation, can enhance biochar functionality for sustainable soil management and crop production.

## 2. Results

### 2.1. Changes in pH and EC

**Figure 1:**
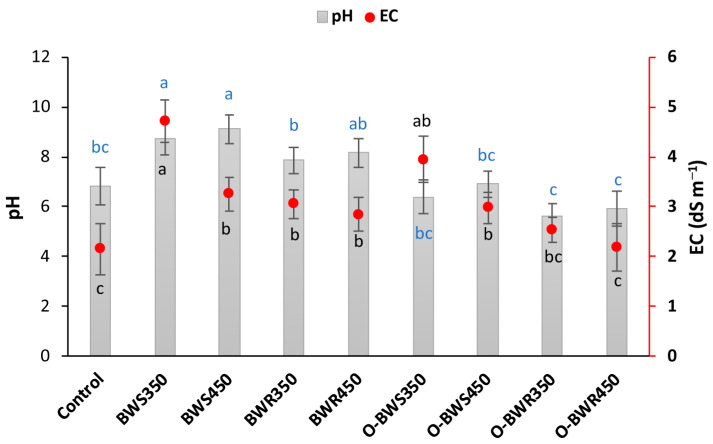
Effect of biochar variations on soil pH and EC. Biochars’ abbreviations are defined as follows: BWS: wheat straw, and BWR: wood residues. Prefix O- refers to oxidized biochar, and suffixes 350 and 450 refer to pyrolysis temperatures during biochar production. Values are presented as mean ± standard error (SE) of three replicates. Lower cases represent the significant difference among treatments.

Biochar application significantly affected soil pH and electrical conductivity (EC) compared with the control (Figure 1). The control soil had an initial pH of 6.82. Pristine biochars markedly increased soil pH, with the highest values recorded in BWR_350_ (8.73) and BWR_450_ (9.12). This suggests that wood residue biochars exerted a stronger liming effect than wheat straw biochars. In contrast, oxidized biochars lowered soil pH, with the minimum values observed in O-BWS_350_ (5.62) and O-BWS_450_ (5.93). Similarly, biochar addition significantly increased soil EC, with the highest value measured in BWS_350_, followed by other pristine biochars.

### 2.2. Changes in PO_4_^3−^ Leaching

**Figure 2:**
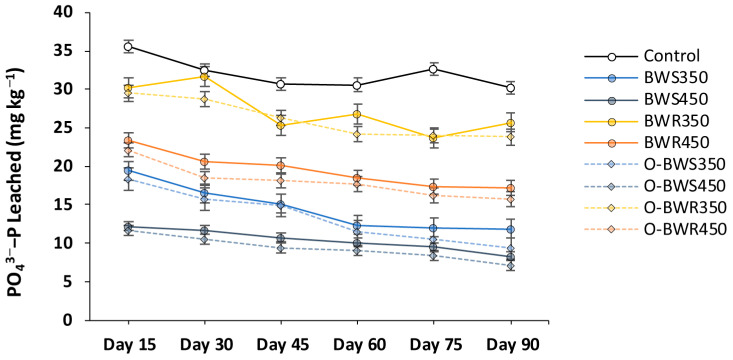
Changes in PO_4_^3−^-P (mg kg^−1^ soil) leaching dynamics over the experiment period. Biochars’ abbreviations are defined as follows: BWS: wheat straw, and BWR: wood residues. Prefix O- refers to oxidized biochar, and suffixes 350 and 450 refer to pyrolysis temperatures during biochar production. Values are presented as mean ± standard error (SE) of three replicates.

Biochar application significantly reduced PO_4_^3−^-P leaching compared with the control (Figure 2). Across all treatments, PO_4_^3−^-P concentrations declined over time, indicating progressive nutrient stabilization. Biochars produced at higher pyrolysis temperatures (450 °C) were generally more effective than those produced at 350 °C. The lowest PO_4_^3−^-P leaching was observed in O-BWS_450_ (9.39 mg kg^−1^), representing a 70.7% reduction compared with the control (32.07 mg kg^−1^), followed by O-BWS_350_ (13.38 mg kg^−1^; 58.3% reduction). Oxidized wood biochars also reduced P leaching, with O-BWR_450_ at 18.04 mg kg^−1^ (43.7% reduction) and O-BWR_350_ at 26.10 mg kg^−1^ (18.5% reduction), although their effect was less pronounced than that of wheat straw biochars. Overall, oxidation enhanced P retention across feedstocks, but differences between pristine and oxidized forms were not always statistically significant.

### 2.3. Changes in NO_3_^−^ and NH_4_^+^ Leaching

**Figure 3:**
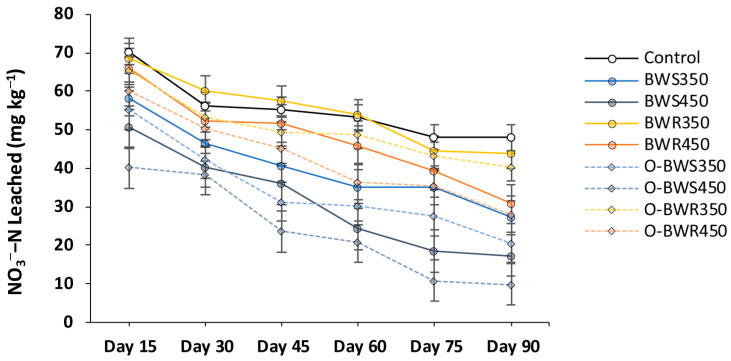
Changes in NO_3_^−^-N (mg kg^−1^ soil) leaching dynamics over the experiment period. Biochars’ abbreviations are defined as follows: BWS: wheat straw, and BWR: wood residues. Prefix O- refers to oxidized biochar, and suffixes 350 and 450 refer to pyrolysis temperatures during biochar production. Values are presented as mean ± standard error (SE) of three replicates.

**Figure 4:**
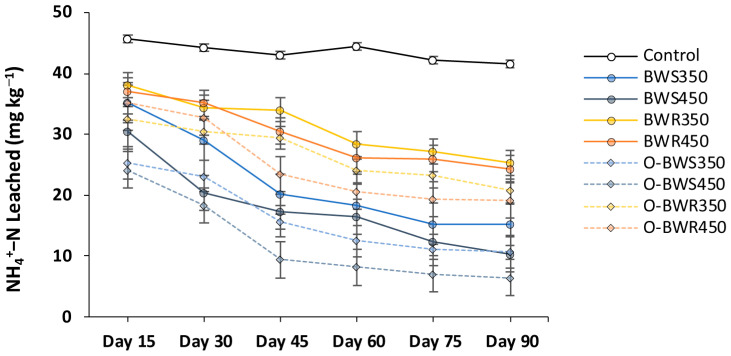
Changes in NH_4_^+^-N (mg kg^−1^ soil) leaching dynamics over the experiment period. Biochars’ abbreviations are defined as follows: BWS: wheat straw, and BWR: wood residues. Prefix O- refers to oxidized biochar, and suffixes 350 and 450 refer to pyrolysis temperatures during biochar production. Values are presented as mean ± standard error (SE) of three replicates.

Both NO_3_^−^-N and NH_4_^+^-N leaching decreased over time across all biochar treatments (Figure 3 and Figure 4). Wood residue biochars (BWR_350_ and BWR_450_) reduced NO_3_^−^-N leaching compared with the control, although the effect became significant only after 60 days. The strongest reductions occurred with oxidized wheat straw biochars, particularly O-BWS_450_, which lowered NO_3_^−^-N concentrations from 70.32 mg kg^−1^ (control) to 47.99 mg kg^−1^ at day 15 (31.7% reduction) and further down to 9.65 mg kg^−1^ at day 90 (86.3% reduction).

A similar pattern was observed for NH_4_^+^-N leaching. The control remained relatively steady at an average of 43.52 mg kg^−1^, while all biochar treatments significantly reduced NH_4_^+^-N losses. The lowest NH_4_^+^-N concentrations were recorded in O-BWS_450_ (12.24 mg kg^−1^; 71.9% reduction) and O-BWS_350_ (16.41 mg kg^−1^; 62.3% reduction), followed by their pristine counterparts BWS_450_ (17.86 mg kg^−1^; 58.9% reduction) and BWS_350_ (22.23 mg kg^−1^; 48.9% reduction). Overall, oxidation and higher pyrolysis temperature enhanced the ability of biochars to retain inorganic nitrogen and limit leaching.

### 2.4. Changes in N and P Uptakes in the Plant

Biochar application significantly improved both nitrogen (N) and phosphorus (P) uptake in fava bean compared with the control (Table 1). The control plants had the lowest uptake values (N: 3.65%; P: 2.35%). Among the pristine biochars, BWS_450_ achieved the highest N (5.13%; 1.4-fold increase, 40.3% higher than control) and P (2.67%; 1.14-fold increase, 13.6% higher than control) uptake, followed closely by BWR_450_ (N: 4.94%; 1.35-fold, 35.3% increase; P: 2.54%; 1.08-fold, 8.1% increase).

Oxidized biochars further enhanced nutrient uptake. The greatest N and P uptakes were observed in O-BWS_450_ (N: 6.71%; 1.84-fold, 83.8% increase; P: 3.89%; 1.66-fold, 65.5% increase), followed by O-BWR_450_ (N: 6.07%; 1.66-fold, 66.3% increase; P: 3.43%; 1.46-fold, 46.0% increase) and O-BWS_350_ (N: 5.42%; 1.48-fold, 48.5% increase; P: 3.52%; 1.50-fold, 49.8% increase). Overall, oxidized wheat straw biochars outperformed all other treatments, highlighting the synergistic effects of feedstock type, pyrolysis temperature, and surface oxidation on improving nutrient uptake.

Biochars’ abbreviations are defined as follows: BWS: wheat straw, and BWR: wood residues. Prefix O- refers to oxidized biochar, and suffixes 350 and 450 refer to pyrolysis temperatures during biochar production. Values are presented as mean ± standard error (SE) of three replicates.

## 3. Discussion

### 3.1. Changes in pH and EC

The increase in pH with pristine biochars can be attributed to the presence of alkaline mineral ash (Ca, Mg, K, Na, carbonates, and oxides), which neutralizes soil acidity [27,28]. The stronger effect of wheat straw-derived biochars likely reflects their higher ash alkalinity compared to wood biochars. The higher nutrient content in straw-based herbaceous materials yields biochar, which was further supported by several previous studies [29,30]. In general, wood-derived biochars tend to exhibit pH values that are about two units lower than those produced from other biomass sources under comparable pyrolysis conditions [31]. The elevation in pH during pyrolysis is largely associated with the enrichment of non-pyrolyzed mineral constituents and the breakdown of the organic matrix [32]. The results of biochar characterizations in Table 3 also clearly show the higher ash content in wheat straw biochar (13.83–16.42%) than wood biochar (7.14–8.49%). In contrast, oxidation with H_2_O_2_ introduced acidic functional groups (e.g., carboxyl and phenolic groups) on the biochar surface, which contributed to lowering soil pH [33,34]. Similar findings have been reported where surface oxidation reduced the liming capacity of biochars [28,35,36]. Changes in soil pH are highly significant because pH is a master variable that affects numerous physical, chemical, and biological soil processes. Increases in pH, as observed with pristine biochars, can enhance the availability of essential nutrients such as calcium, magnesium, and molybdenum, while potentially reducing the solubility of metals like iron, manganese, and zinc [37]. Elevated pH can also influence microbial activity by favoring bacteria over fungi and modifying enzymatic processes that drive nutrient cycling [38]. Additionally, shifts in pH can alter soil structure by affecting aggregate stability and cation exchange interactions, which in turn influence water retention and root penetration [39]. Conversely, the slight acidification observed with oxidized biochars may increase the solubility of some nutrients (e.g., phosphorus) but could also affect microbial communities differently. Overall, these pH-mediated effects highlight the broader implications of biochar application for soil fertility, plant growth, and ecosystem functioning.

Differences between pristine and oxidized biochars were less pronounced, as oxidation did not markedly alter EC relative to their corresponding untreated forms. The elevated EC following biochar addition is consistent with the release of soluble salts from the biochar structure [40,41]. Low-temperature biochars, particularly BWS_350_, contain more soluble organic and inorganic compounds, explaining their higher EC [42]. The lack of large differences between pristine and oxidized biochars suggests that oxidation altered functional chemistry more than the salt content, thereby exerting less influence on EC. Notably, the rise in EC observed with biochar application could influence soil salinity and crop performance [40]. Increased EC may create osmotic stress, limiting plant water uptake and reducing nutrient absorption, which can negatively affect growth [43]. Elevated soluble salts can also disrupt soil microbial communities, potentially altering nutrient cycling and moderating the positive effects of biochar on soil fertility [44]. Therefore, while biochar improves nutrient retention and availability, it is important to monitor EC levels, particularly in soils susceptible to salinization, to ensure that the benefits are not offset by salinity-related risks.

### 3.2. Changes in PO_4_^3−^ Leaching

The reduction in PO_4_^3−^-P leaching following biochar addition is likely due to the increased sorption capacity of soil through mechanisms such as ligand exchange [45], electrostatic attraction [46], and precipitation with metal cations (Ca^2+^, Mg^2+^, Fe^3+^, and Al^3+^) [47]. Biochars produced at higher pyrolysis temperatures (450 °C) typically exhibit greater surface area and aromaticity, enhancing phosphate adsorption and thereby reducing leaching [48,49]. The stronger effect of oxidized biochars, particularly those derived from wheat straw, can be attributed to the introduction of oxygen-containing functional groups (–COOH, –OH) that provide additional binding sites for phosphate [24]. These findings are consistent with earlier reports that surface oxidation enhances biochar’s P-retention capacity, although the extent of reduction varies with feedstock type [50,51,52].

### 3.3. Changes in NO_3_^−^ and NH_4_^+^ Leaching

The reduction of NO_3_^−^-N and NH_4_^+^-N leaching through biochar application can be attributed to multiple mechanisms. Biochars, particularly those produced from wheat straw at lower temperatures, contain more labile organic compounds and surface functional groups that enhance cation and anion retention [5]. Oxidized biochars further improve this capacity through the introduction of additional oxygen-containing groups (–COOH, –OH), which increase cation exchange capacity (CEC) for NH_4_^+^ and anion binding for NO_3_^−^ [53,54]. The superior performance of O-BWS_450_ suggests that the combination of moderate pyrolysis temperature (providing structural stability and surface area) and oxidation (introducing sorption sites) optimizes nutrient retention. The delayed effect of BWR biochars indicates their lower initial nutrient availability but stronger, longer-term stabilization capacity, consistent with their denser structure and higher aromaticity [10]. Overall, these results indicate that wheat straw-derived oxidized biochars, particularly O-BWS_450_, offer the best balance between reducing both nitrate and ammonium leaching, thereby improving nutrient use efficiency and minimizing environmental losses.

### 3.4. Changes in N and P Uptakes in the Plant

The improvement in N and P uptake under biochar treatments can be attributed to multiple mechanisms. First, biochars reduced nutrient leaching (as shown in Section 3.2 and Section 3.3), leading to greater nutrient availability for plant uptake. Previous studies have shown that iron-enriched biochar exhibits superior sorption capacity for NO_3_^−^, PO_4_^3−^, and NH_4_^+^ compared to unmodified biochar. This enhancement helps to minimize nutrient leaching from soil while simultaneously improving nutrient availability for plant uptake [55]. Second, the higher surface area and porosity of oxidized biochars, particularly those produced at 450 °C, improved nutrient retention and release to plant roots. Oxidation also introduced functional groups that enhanced interactions with N and P species, reducing losses while making them more accessible to plants [22]. The superior performance of O-BWS_450_ highlights the synergistic effect of feedstock type, pyrolysis temperature, and surface modification. Wheat straw biochars, being richer in nutrients and more labile organic matter, provided readily available P and enhanced rhizosphere interactions [56].

It is important to note that while biochar sorbs N and P, reducing their mobility in the soil solution, this does not permanently immobilize the nutrients. Instead, biochar acts as a nutrient reservoir, gradually releasing N and P in the root zone. This mechanism explains the observed relationship: increased sorption by biochar → decreased leaching → more nutrients retained in the root zone → increased N and P uptake by plants. By retaining nutrients where roots can access them, biochar synchronizes nutrient availability with plant demand, enhancing overall nutrient use efficiency.

In general, the addition of biochar can enhance plant phosphorus uptake by influencing soil phosphatase activity and stimulating the activity of mycorrhizal fungi. Experiments have demonstrated that varying amounts of biochar can inhibit acid phosphatase activity while enhancing that of alkaline phosphatase, promoting the hydrolysis of PO_4_^3−^ and ensuring a continuous supply of available P in the soil [57,58]. At the same time, oxidation amplified sorption capacity and nutrient exchange, leading to improved uptake [59,60]. These findings align with previous studies reporting that biochar surface oxidation enhances nutrient use efficiency in plants [61,62]. Although morphological parameters such as plant height, leaf area, or root traits were not measured in this experiment, the observed increases in aboveground biomass and N and P uptake indicate substantial improvements in plant performance under biochar treatments. Future studies could include these additional morphological traits to provide a more comprehensive understanding of biochar effects on plant growth. It is also important to note that soil microbial community structure and enzymatic activity were not assessed in this experiment due to laboratory limitations. These factors can play a crucial role in nutrient cycling and biochar-mediated soil fertility, and their inclusion could provide a deeper mechanistic understanding of biochar effects on soil–plant interactions.

## 4. Materials and Methods

### 4.1. Experimental Setup

The study was carried out in a greenhouse experiment scale at the Department of Agroecosystems, Faculty of Agriculture and Technology, University of South Bohemia (České Budějovice, Czech Republic) to investigate the effects of pristine and oxidized biochars on soil chemical properties and fava bean (*Vicia faba* L.) performance. The soil used in the experiment was collected in September 2023 from the top 0–20 cm layer of an arable field belonging to the university’s Faculty of Agriculture and Technology (48°58′29.8″ N, 14°26′55.8″ E). The soil had a loam texture (44.8% sand, 24.1% silt, 31.1% clay) and an initial pH of 6.82. After air-drying and sieving (<5 mm), the soil was thoroughly mixed with the assigned biochar treatment and used to fill plastic pots containing 3 kg of soil each.

The experiment included nine treatments: four pristine biochars, four oxidized biochars, and one unamended control, each replicated three times (total of 27 pots). The experiment followed a completely randomized design (CRD), and each treatment was randomly assigned to the pots to minimize positional effects within the greenhouse.

A basal dose of NPK 20:20:20 fertilizer (100 mg N kg^−1^ soil, applied proportionally as 0.5 g fertilizer per kg soil) was added uniformly to all pots at the time of planting. No further fertilization was conducted during the experiment. This low-level application allowed monitoring of nutrient leaching and plant uptake while isolating the effects of biochar treatments.

Fava bean seeds were used as the test crop. Three seeds were sown per pot, and after two weeks of emergence, the two weakest seedlings in each pot were removed to leave the most vigorous plant. The pots were maintained under controlled greenhouse conditions with a temperature of 25 ± 2 °C during the day and 20 ± 2 °C at night, relative humidity of 50–70%, and a 16 h light/8 h dark photoperiod. Soil moisture was monitored weekly and adjusted to maintain approximately 30% above field capacity. Field capacity was determined by saturating a soil subsample, allowing it to drain for 24 h, and weighing it. Pots were initially watered to reach the target moisture and then weighed weekly. The water lost through evaporation and transpiration was replenished with distilled water to maintain consistent soil moisture throughout the 90-day experiment. The experiment duration of 90 days was selected to cover the key vegetative and early reproductive stages of fava bean growth, allowing sufficient time to observe biochar effects on soil chemical properties, nutrient leaching, and plant nutrient uptake. This period also ensures measurable changes in soil pH, EC, and nutrient dynamics under greenhouse conditions. At the end of the period, plant and soil samples were collected for further analyses.

### 4.2. Biochar Production and Modification

Two biomass feedstocks—wheat straw (WS) and wood residue (WR)—were selected for biochar production. Each was subjected to pyrolysis at 350 °C and 450 °C in an electric muffle furnace (Nabertherm Medlin, Lilienthal, Germany), using a consistent heating rate of 10 °C per minute, a residence time of 2 h, and nitrogen gas to maintain an oxygen-limited environment. Post-pyrolysis, biochars were rinsed twice with deionized water to remove impurities, oven-dried at 105 °C for 24 h. Each biochar sample was then split into two subgroups: one kept as unmodified (pristine) and the other subjected to oxidation using hydrogen peroxide (H_2_O_2_).

For oxidation, approximately 10 g of biochar was placed in 50 mL of 10% H_2_O_2_ solution and stirred at 70 °C for 2 h in a 100 mL glass beaker. The samples were then filtered (Whatman No. 42), rinsed thoroughly with deionized water, and dried again at 105 °C for 24 h. This resulted in a total of eight biochar treatments: pristine biochars: BWS_350_, BWS_450_, BWR_350_, BWR_450_, and oxidized biochars: O-BWS_350_, O-BWS_450_, O-BWR_350_, O-BWR_450_. A summary of treatments and combinations is presented in Table 2.

Each biochar type was applied to soil at a rate of 2% *w*/*w*, thoroughly mixed, and used to fill the experimental pots. A control treatment without biochar was included. All treatments were conducted in triplicate.

### 4.3. Biochar Characterization

The physical and chemical properties of the eight biochar variants were analyzed before their application. Biochar pH and EC were measured in a 1:10 (*w*/*v*) biochar-to-distilled water suspension. Total carbon, hydrogen, and nitrogen contents were determined using a CHN elemental analyzer (TruSpec Micro CHN, Leco, St. Joseph, MI, USA). The cation exchange capacity (CEC) was analyzed using the ammonium acetate extraction method [24]. Total porosity (V_t_) of biochar samples was measured with a mercury porosimetry AutoPore IV 9500 M (Micromeritics, Norcross, GA, USA). To analyze the specific surface area (SSA) of biochar samples, the Brunauer–Emmett–Teller (BET) method was conducted with nitrogen adsorption/desorption isotherms obtained at 196 °C using the ASAP 2420 apparatus (Micromeritics, GA, USA). The results of biochar analysis are provided in Table 3. Further morphological and structural characterizations (e.g., SEM and FTIR) of these biochars can be found in our already published work [24].

**Table 3:** Biochar physiochemical properties.

	pH	EC (dS m^−1^)	CEC (cmol^+^ kg^−1^)	C (%)	H (%)	N (%)	O (%)	VM (%)	AC (%)	SSA (m^2^ g^−1^)	V_t_ (cm^3^ g^−1^)
BWS_350_	7.04	1.97	62.7	55.4	4.32	2.31	21.2	46.7	16.42	103.9	0.031
BWS_450_	7.75	2.12	52.8	65.8	3.72	1.24	15.1	34.6	13.83	132.4	0.037
BWR_350_	8.41	1.06	45.7	68.5	4.23	1.36	18.3	37.1	7.14	89.62	0.019
BWR_450_	8.89	1.11	34.8	72.8	3.84	1.15	13.3	32.9	8.49	96.81	0.023
O-BWS_350_	4.22	1.91	74.2	52.2	4.24	1.38	26.3	42.1	15.6	136.1	0.095
O-BWS_450_	4.68	2.16	68.4	62.6	2.13	1.29	17.2	29.8	16.59	159.8	0.125
O-BWR_350_	5.34	1.09	43.5	66.8	3.94	0.95	18.2	28.4	9.74	113.7	0.094
O-BWR_450_	6.63	1.22	34.6	75.5	3.18	0.66	12.3	25.1	8.07	158.4	0.116

EC: electric conductivity, CEC: cation exchange capacity, C: carbon, H: hydrogen, N: nitrogen, O: oxygen, VM: volatile matter, AC: ash content, SSA: specific surface area, V_t_: total pore volume. Biochars’ abbreviations are defined as follows: BWS: wheat straw, and BWR: wood residues. Prefix O- refers to oxidized biochar, and suffixes 350 and 450 refer to pyrolysis temperatures during biochar production.

### 4.4. Soil and Plant Analyses

At the end of the 90-day experiment, soil samples were collected from each pot to evaluate soil pH and electrical conductivity (EC). Soil pH and EC were measured in a 1:2.5 (*w*/*v*) soil-to-distilled water suspension. Soil pH was determined using a calibrated glass electrode pH meter (pH 340, WTW, Weilheim, Germany), and EC was measured with a conductivity meter (Cond 3310, WTW, Weilheim, Germany). To monitor changes in nutrient leaching over time, leachate samples were collected from the bottom of the pots immediately after irrigation events at six time points: on Day 0 (immediately after planting) and then every 15 days throughout the experiment. The collected leachates were filtered through Whatman No. 42 filter paper and analyzed for nitrate (NO_3_^−^-N) and ammonium (NH_4_^+^-N) using a continuous flow analyzer (Skalar San++, Skalar Analytical, The Netherlands). Phosphate (PO_4_^3−^-P) concentrations were determined colorimetrically using the molybdenum blue method with spectrophotometric detection (UV-1800, Shimadzu, Kyoto, Japan) at 880 nm.

At the end of the experiment, aboveground parts of the fava bean plants were harvested by cutting the stems 2 cm above the soil surface. The harvested biomass was oven-dried at 65 °C until constant weight and then weighed to determine aboveground dry biomass. Dried plant tissues were finely ground and analyzed for total nitrogen and phosphorus content using the Kjeldahl digestion and molybdenum-blue colorimetric method, respectively. From these values, N and P uptake per plant was calculated by multiplying nutrient concentration by the corresponding biomass weight.

### 4.5. Data Analyses

All experimental data were analyzed using R software (version 4.4.1). The normality and homogeneity of variance of the data were tested using the Shapiro–Wilk and Levene’s tests, respectively. Where necessary, data were log-transformed to meet the assumptions of parametric analysis.

A one-way analysis of variance (ANOVA) was performed to evaluate the effects of biochar treatments on soil properties, nutrient leaching, and nutrient uptake. When significant differences (*p* < 0.05) were detected, means were compared using Tukey’s honestly significant difference (HSD) post hoc test. Graphical representations of data were created using the ggplot2 package in R.

## 5. Conclusions

This study demonstrated that both feedstock type, pyrolysis temperature, and post-production oxidation strongly influence biochar performance in soil–plant systems. Pristine biochars increased soil pH and electrical conductivity, with wheat straw biochars producing greater alkalinity, whereas oxidized biochars reduced soil pH due to the introduction of oxygen-containing functional groups. Across treatments, biochars effectively reduced nutrient leaching, with oxidized wheat straw biochar produced at 450 °C (O-BWS_450_) showing the greatest efficiency in retaining NO_3_^−^-N, NH_4_^+^-N, and PO_4_^3−^-P. Enhanced nutrient retention translated into improved plant nutrient uptake. Fava bean plants grown in soils amended with oxidized biochars exhibited significantly higher N and P uptake, with O-BWS_450_ outperforming all other treatments. These findings highlight the synergistic effects of pyrolysis temperature and oxidation in enhancing biochar’s capacity to improve soil nutrient dynamics and plant nutrition. Overall, the results suggest that surface-modified biochars, particularly oxidized wheat straw biochar at 450 °C, hold strong potential as soil amendments to improve nutrient availability and reduce environmental losses. The novelty of this work lies in systematically evaluating multiple feedstocks, pyrolysis temperatures, and oxidation treatments under a controlled soil–plant system, providing mechanistic insights into how biochar engineering can optimize nutrient retention and plant nutrition. Future studies should validate these findings under field conditions, incorporate soil enzymatic and microbial analyses, include plant morphological traits, and explore additional biochar feedstocks and modifications to develop optimized, feedstock-specific biochars for sustainable agriculture.

## Figures and Tables

**Table 1:** Effect of pristine and modified biochar on nitrogen and phosphorus uptake in fava bean plant.

	N-Uptake (%)	P-Uptake (%)
Control	3.65 ± 0.13 c	2.35 ± 0.11 c
BWS_350_	4.77 ± 0.21 bc	2.41 ± 0.08 bc
BWS_450_	5.13 ± 0.75 ab	2.67 ± 0.18 b
BWR_350_	4.64 ± 0.24 bc	2.38 ± 0.12 bc
BWR_450_	4.94 ± 0.36 b	2.54 ± 0.17 bc
O-BWS_350_	5.42 ± 0.48 ab	3.52 ± 0.33 a
O-BWS_450_	6.71 ± 0.53 a	3.89 ± 0.47 a
O-BWR_350_	5.25 ± 0.39 ab	2.75 ± 0.16 b

Lower cases represent the significant difference among treatments.

**Table 2:** Biochar treatment combinations used in the experiment.

	Feedstock	Pyrolysis Temp (°C)	Oxidation Treatment	Description
BWS_350_	Wheat straw	350	None	Pristine wheat straw biochar
BWS_450_	Wheat straw	450	None	Pristine wheat straw biochar
BWR_350_	Wood residue	350	None	Pristine wood biochar
BWR_450_	Wood residue	450	None	Pristine wood biochar
O-BWS_350_	Wheat straw	350	H_2_O_2_	Oxidized wheat straw biochar
O-BWS_450_	Wheat straw	450	H_2_O_2_	Oxidized wheat straw biochar
O-BWR_350_	Wood residue	350	H_2_O_2_	Oxidized wood biochar
O-BWR_450_	Wood residue	450	H_2_O_2_	Oxidized wood biochar

## Data Availability

Data are contained within the article.
